# Supplementary figures and images for: Fast, Sensitive and Specific Detection of Thailand orthohantavirus and Its Variants Using One-Step Real-Time Reverse-Transcription Polymerase Chain Reaction Assay
Source: Viruses. 2019 Aug 6;11(8):718. doi: 10.3390/v11080718 (PMC6722858; doi:10.3390/v11080718)

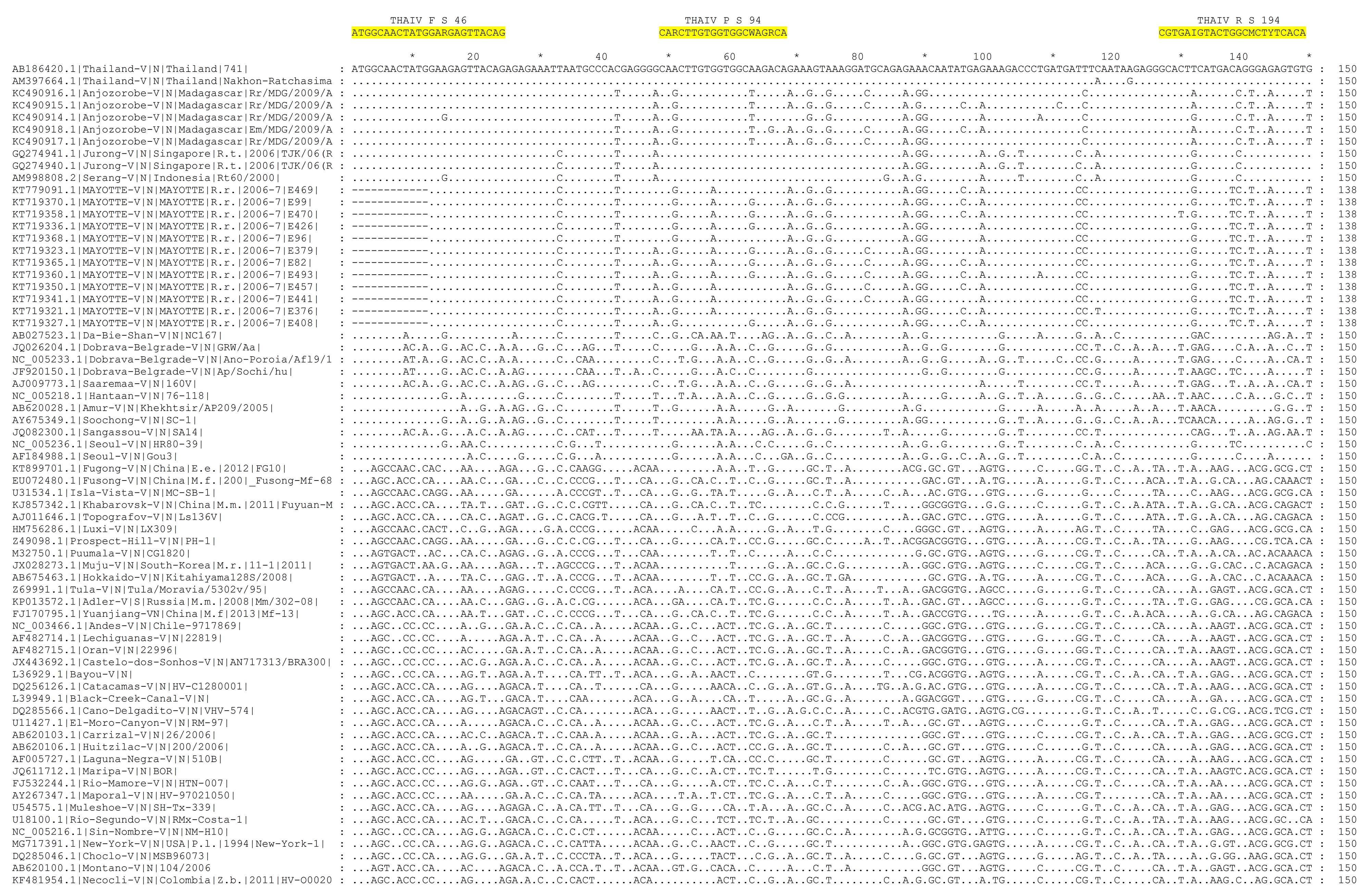

Supplement: Supplementary file 1 [file viruses-11-00718-s001.zip › Viruses-526117_Figure S1.jpg]
